# Bis(morpholin-4-ium) tetra­chlorido­cobalt(II)

**DOI:** 10.1107/S1600536811053128

**Published:** 2011-12-14

**Authors:** Wen-Zhen Wang, Rayyat Huseyn Ismayilov, Gene-Hsiang Lee, Yuh-Sheng Wen, Shie-Ming Peng

**Affiliations:** a School of Chemistry and Chemical Engineering, Xi’an Shiyou University, Xi’an 710065, People’s Republic of China; b Institute of Chemical Problems, Azerbaijan Academy of Sciences, Baku, Azerbaijan; c Instrumentation Center, National Taiwan University, Taipei 106, Taiwan; d Institute of Chemistry, Academia Sinica, Taipei 106, Taiwan; e Department of Chemistry, National Taiwan University, Institute of Chemistry, Academia Sinica, Taipei 106, Taiwan

## Abstract

The title compound, (C_4_H_10_NO)_2_[CoCl_4_], is an ionic compound consisting of two protonated tetra­hydro-1,4-oxazine (morpholine) cations and a [CoCl_4_]^2−^ dianion. The Co^II^ ion is in a tetra­hedral coordination geometry. The cations exhibit chair-shaped conformations. A three-dimensional supra­molecular architecture is formed through N—H⋯Cl and C—H⋯Cl hydrogen bonds between the dianions and the cations.

## Related literature

For background to this class of compound, see: Ismayilov *et al.* (2007 ▶); Kiehl *et al.* (2004 ▶); Leung *et al.* (2002 ▶); Wang *et al.* (2007 ▶, 2008 ▶). For the synthesis, see: Wang *et al.* (2007 ▶, 2008 ▶). For related structures, see: Fastje & Möller (2009 ▶); Szklarz *et al.* (2009 ▶); Wu *et al.* (1997 ▶).
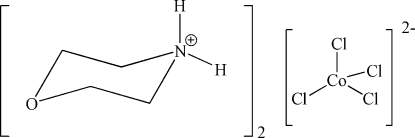


## Experimental

### 

#### Crystal data

(C_4_H_10_NO)_2_[CoCl_4_]
*M* *_r_* = 376.99
Monoclinic, 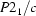

*a* = 9.7545 (5) Å
*b* = 15.0283 (8) Å
*c* = 10.4785 (5) Å
β = 94.064 (3)°
*V* = 1532.22 (13) Å^3^
*Z* = 4
Mo *K*α radiation
μ = 1.81 mm^−1^
*T* = 100 K
0.20 × 0.16 × 0.06 mm

#### Data collection

Bruker SMART APEX CCD area-detector diffractometer
Absorption correction: multi-scan (*SADABS*; Bruker, 2001 ▶) *T*_min_ = 0.714, *T*_max_ = 0.899
10714 measured reflections
2661 independent reflections
2001 reflections with *I* > 2σ(*I*)
*R*_int_ = 0.047

#### Refinement

*R*[*F* ^2^ > 2σ(*F* ^2^)] = 0.030
*wR*(*F* ^2^) = 0.059
*S* = 0.89
2661 reflections
154 parameters
H-atom parameters constrained
Δρ_max_ = 0.32 e Å^−3^
Δρ_min_ = −0.32 e Å^−3^

### 

Data collection: *SMART* (Bruker, 2007 ▶); cell refinement: *SAINT* (Bruker, 2007 ▶); data reduction: *SAINT*; program(s) used to solve structure: *SHELXS97* (Sheldrick, 2008 ▶); program(s) used to refine structure: *SHELXL97* (Sheldrick, 2008 ▶); molecular graphics: *XP* in *SHELXTL* (Sheldrick, 2008 ▶); software used to prepare material for publication: *SHELXTL*.

## Supplementary Material

Crystal structure: contains datablock(s) global, I. DOI: 10.1107/S1600536811053128/pk2366sup1.cif

Structure factors: contains datablock(s) I. DOI: 10.1107/S1600536811053128/pk2366Isup2.hkl

Additional supplementary materials:  crystallographic information; 3D view; checkCIF report

## Figures and Tables

**Table 1:** Hydrogen-bond geometry (Å, °)

*D*—H⋯*A*	*D*—H	H⋯*A*	*D*⋯*A*	*D*—H⋯*A*
N1—H1*A*⋯Cl2^i^	0.92	2.40	3.275 (3)	159
N1—H1*B*⋯Cl1	0.92	2.38	3.184 (2)	146
N2—H2*A*⋯Cl3	0.92	2.45	3.232 (3)	142
N2—H2*B*⋯Cl3^ii^	0.92	2.37	3.264 (3)	163
C2—H2*C*⋯Cl1^ii^	0.99	2.71	3.603 (3)	151
C3—H3*B*⋯Cl4^i^	0.99	2.77	3.556 (3)	136
C5—H5*A*⋯Cl3^iii^	0.99	2.83	3.767 (3)	158

Symmetry codes: (i) 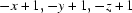; (ii) 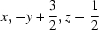; (iii) 
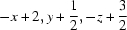
.

**Fig. 1.**
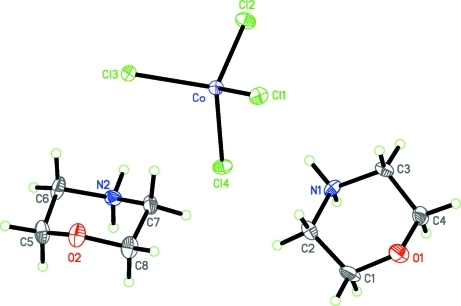
The molecular structure of (I) with ellisoids at the 30% probability level.

**Fig. 2.**
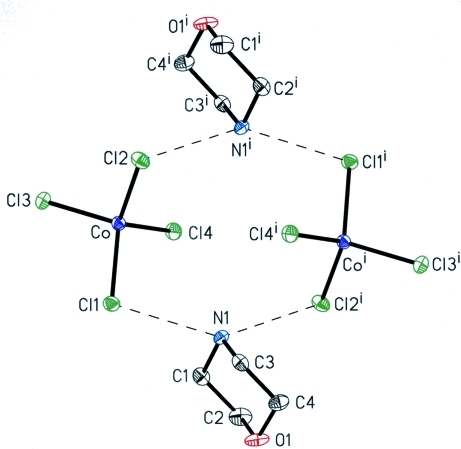
An 8-membered ring generated by hydrogen bonds (dashed lines) in (I). Atoms labelled with the suffixes i are at the symmetry equivalent position (-x + 1, -y + 1, -z + 1). Ellipsoids are drawn at the 30% probability level.

**Fig. 3.**
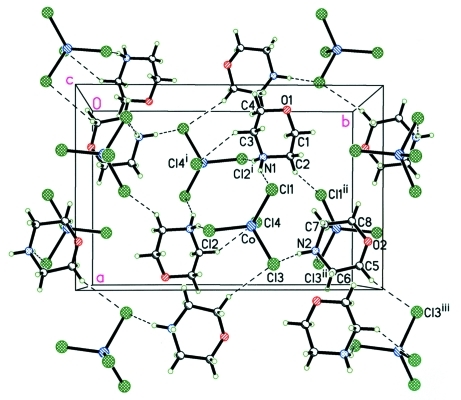
A packing diagram of (I) viewed down the c axis. Dashed lines represent hydrogen bonds. Atoms labelled with the suffixes i, ii, and iii are at symmetry equivalent positions (-x + 1, -y + 1, -z + 1), (x, -y + 3/2, z - 1/2) and (-x + 2, y + 1/2, -z + 3/2), respectively.

## supplementary crystallographic information

### Comment

Oligo-α-pyridylamine ligands are very useful in the synthesis of metal string complexes, which are also known as extended metal atom chains (EMACs).  EMACs are invaluable for acquiring a fundamental understanding of metal-metal bonds (Kiehl *et al.*, 2004) and for potential applications such as molecular electronic devices. These oligo-α-pyridylamine ligands contain pyridyl and amine groups, and can result in the formation of double helical structures in nonpolar solvents due to hydrogen bonding (Leung *et al.*, 2002). After deprotonation of oligo-α-pyridylamine ligands, the resulting anions can stabilize the linear metal cores.  Activation of the H atom was observed in some EMACs. Recently we designed a series of modulated oligo-a-pyridylamino ligands, by including one or more of the nitrogen-rich aromatic rings such as pyrazine, pyrimidine and naphthyridine instead of pyridine rings to the ligands. The modification of ligands significantly improved the reactivity leading to the EMAC, and resulted in complexes with very different redox properties (Wang *et al.*, 2008).  Furthermore, by providing more donor nitrogen atoms in aromatic rings, the pyrazine ligands exhibit more coordination forms and are especially versatile in the construction of coordination polymers with potential applications in gas storage, catalysis, magnetism, luminescence, *etc*. due to their ability to form multidimensional frameworks through multiple metal-binding sites. (Ismayilov *et al.*, 2007; Wang *et al.*, 2007, 2008). Some interesting phenomena were also observed, *e.g.* the observation of disassembly of ligands during the preparation of EMACs. In this paper we describe a compound (I) from the decomposition of pyrazine-modulated *N*^2^-(pyrazin-2-yl)-*N*^6^– (6-(pyrazin-2-ylamino)pyridin-2-yl)pyridine-2,6-diamine (H_3_pzpz) relating to the preparation of heptacobalt complexes (Wang *et al.*, 2007).

The crystal structure of [C_4_H_10_NO]_2_[CoCl_4_] shown in Fig. 1 consists of two protonated tetrahydro-1,4-oxazine(morpholine) cations [C_4_H_10_NO]^+^ and a [CoCl_4_]^2-^ dianion, the compound [C_4_H_10_NO]_2_[CoCl_4_] is a hybrid ionic inorganic-organic compound of Y_2_*X* type. The Co^II^ ion is in a tetrahedral coordination geometry. The Co—Cl bond distances are in the range 2.2583 (8) - 2.2818 (8)Å with an average of 2.2675 (8)Å (The Co—Cl in CoCl_2_ is 2.43 Å). The bond angles between Co—Cl are in the range 106.01 (3) - 113.73 (3)° with an average of 109.43 (3)°, which is very close to the ideal tetrahedral angle value of 109.28° (Fastje & Möller, 2009; Szklarz *et al.*, 2009; Wu *et al.*, 1997).

The cations are six-membered heterocycle rings, protonated tetrahydro- 1,4-oxazine, and exhibit chair-shaped conformations. All bond angles in the rings are in the range 108.8 (3) - 111.9 (2)° with an average of 110.4 (3)°, which is well consistent with a *sp*^3^ hybrid orbital angle. The nitrogen atom in tetrahydro-1,4-oxazine was protonated owing to the low basicity of tetrahydro-1,4-oxazine (pK_a_ = 8.4) due to the inductive effect of oxygen atom. The average C—C, C—N and C—O bond distances exhibit a typical value of single bonds, which are 1.503 (4), 1.490 (4) and 1.419 (4) Å, respectively.

Extensive hydrogen bonds between chloride atoms Cl(1), Cl(2) and Cl(3) in the [CoCl_4_]^2-^ dianion and nitrogen and oxygen atoms N(1), N(2), O(1) and O(2) in both tetrahydro-1,4-oxazine cations were observed (Table 1). [CoCl_4_]^2-^ dianions were paired through the hydrogen bonds between Cl(1), Cl(2) and N(1) atoms, resulting in an 8-membered ring N(1)···Cl(1)—Co—Cl(2)···N(1)···Cl(1)—Co—Cl(2) (Fig. 2) which were further linked to a 2-D sheet extending in the *bc* plane through hydrogen bonds between Cl(3) and N(2) atoms. A group of weak hydrogen bonds involving carbon atoms were observed between layers, which build the title compound (I) into a 3-D network (Fig. 3).

### Experimental

Anhydrous CoCl_2_ (254 mg, 1.95 mmol), H_3_pzpz (300 mg, 0.84 mmol) and naphthalene (65 g) were placed in an Erlenmeyer flask. The mixture was heated under argon and then a solution of potassium *tert*-butoxide (311 mg, 2.77 mmol) in *n*-butyl alcohol (5 ml) was added dropwise. The reaction was continued for another 12 h. After cooling the product was transferred to hexane to wash out the remaining naphthalene, and then 100 ml *ca* CH_2_Cl_2_ was used to extract the complex.  A dark green product, mainly the heptacobalt(II) metal string complex, [Co_7_(µ_7_-pzpz)_4_Cl_2_], was obtained after evaporation.  The title compound was obtained as a side product from the reaction.  Light blue single crystals suitable for X-ray diffraction were obtained by diffusion of ether into a chloroform solution of the green product.

### Refinement

H atoms attached to C and N atoms were positioned geometrically and refined using a riding model, with C—H = 0.99 Å, N—H = 0.92Å and *U*_iso_(H) = 1.2Ueq(C,*N*).

### Crystal data

**Table:**

(C_4_H_10_NO)_2_[CoCl_4_]	*F*(000) = 772
*M**_r_* = 376.99	*D*_x_ = 1.634 Mg m^−^^3^
Monoclinic, *P*2_1_/*c*	Mo *K*α radiation, λ = 0.71073 Å
Hall symbol: -P 2ybc	Cell parameters from 2047 reflections
*a* = 9.7545 (5) Å	θ = 2.4–24.0°
*b* = 15.0283 (8) Å	µ = 1.81 mm^−^^1^
*c* = 10.4785 (5) Å	*T* = 100 K
β = 94.064 (3)°	Prism, blue
*V* = 1532.22 (13) Å^3^	0.20 × 0.16 × 0.06 mm
*Z* = 4	

### Data collection

**Table:**

Bruker SMART APEX CCD area-detector diffractometer	2661 independent reflections
Radiation source: fine-focus sealed tube	2001 reflections with *I* > 2σ(*I*)
graphite	*R*_int_ = 0.047
φ and ω scans	θ_max_ = 25.0°, θ_min_ = 3.1°
Absorption correction: multi-scan (*SADABS*; Bruker, 2001)	*h* = −11→10
*T*_min_ = 0.714, *T*_max_ = 0.899	*k* = −17→17
10714 measured reflections	*l* = −12→12

### Refinement

**Table:**

Refinement on *F*^2^	Primary atom site location: structure-invariant direct methods
Least-squares matrix: full	Secondary atom site location: difference Fourier map
*R*[*F*^2^ > 2σ(*F*^2^)] = 0.030	Hydrogen site location: inferred from neighbouring sites
*wR*(*F*^2^) = 0.059	H-atom parameters constrained
*S* = 0.89	*w* = 1/[σ^2^(*F*_o_^2^) + (0.012*P*)^2^ + 2.9504*P*] where *P* = (*F*_o_^2^ + 2*F*_c_^2^)/3
2661 reflections	(Δ/σ)_max_ = 0.001
154 parameters	Δρ_max_ = 0.32 e Å^−^^3^
0 restraints	Δρ_min_ = −0.32 e Å^−^^3^

### Special details

**Table:**

Geometry. All e.s.d.'s (except the e.s.d. in the dihedral angle between two l.s. planes) are estimated using the full covariance matrix. The cell e.s.d.'s are taken into account individually in the estimation of e.s.d.'s in distances, angles and torsion angles; correlations between e.s.d.'s in cell parameters are only used when they are defined by crystal symmetry. An approximate (isotropic) treatment of cell e.s.d.'s is used for estimating e.s.d.'s involving l.s. planes.
Refinement. Refinement of *F*^2^ against ALL reflections. The weighted *R*-factor *wR* and goodness of fit *S* are based on *F*^2^, conventional *R*-factors *R* are based on *F*, with *F* set to zero for negative *F*^2^. The threshold expression of *F*^2^ > σ(*F*^2^) is used only for calculating *R*-factors(gt) *etc*. and is not relevant to the choice of reflections for refinement. *R*-factors based on *F*^2^ are statistically about twice as large as those based on *F*, and *R*- factors based on ALL data will be even larger.

### Fractional atomic coordinates and isotropic or equivalent isotropic displacement parameters (Å^2^)

**Table:**

	*x*	*y*	*z*	*U*_iso_*/*U*_eq_	
Co	0.68020 (4)	0.58131 (3)	0.71185 (4)	0.01425 (11)	
Cl1	0.49824 (8)	0.64671 (5)	0.79526 (7)	0.01891 (18)	
Cl2	0.70544 (8)	0.43579 (5)	0.76441 (7)	0.02066 (19)	
Cl3	0.87551 (8)	0.65335 (5)	0.78680 (7)	0.01802 (18)	
Cl4	0.65461 (8)	0.60624 (5)	0.49825 (7)	0.01990 (19)	
O1	0.0543 (2)	0.71540 (14)	0.4880 (2)	0.0254 (6)	
N1	0.3114 (2)	0.62863 (16)	0.5349 (2)	0.0180 (6)	
H1A	0.3245	0.5998	0.4592	0.022*	
H1B	0.3855	0.6164	0.5914	0.022*	
C1	0.1733 (3)	0.7481 (2)	0.4313 (3)	0.0251 (8)	
H1C	0.1655	0.8134	0.4207	0.030*	
H1D	0.1780	0.7213	0.3454	0.030*	
C2	0.3035 (3)	0.7266 (2)	0.5115 (3)	0.0196 (7)	
H2C	0.3842	0.7463	0.4667	0.024*	
H2D	0.3043	0.7586	0.5942	0.024*	
C3	0.1836 (3)	0.5950 (2)	0.5878 (3)	0.0177 (7)	
H3A	0.1749	0.6202	0.6742	0.021*	
H3B	0.1876	0.5293	0.5954	0.021*	
C4	0.0621 (3)	0.6215 (2)	0.5008 (3)	0.0224 (8)	
H4A	0.0697	0.5943	0.4155	0.027*	
H4B	−0.0230	0.5990	0.5356	0.027*	
O2	0.7598 (2)	0.98715 (13)	0.6677 (2)	0.0219 (5)	
N2	0.8070 (3)	0.81133 (16)	0.5829 (2)	0.0182 (6)	
H2A	0.8219	0.7525	0.6039	0.022*	
H2B	0.8078	0.8167	0.4955	0.022*	
C5	0.8865 (3)	0.9630 (2)	0.6206 (3)	0.0259 (8)	
H5A	0.9605	1.0003	0.6622	0.031*	
H5B	0.8830	0.9745	0.5274	0.031*	
C6	0.9189 (3)	0.8666 (2)	0.6456 (3)	0.0257 (8)	
H6A	1.0076	0.8511	0.6108	0.031*	
H6B	0.9267	0.8550	0.7388	0.031*	
C7	0.6702 (3)	0.8394 (2)	0.6240 (3)	0.0196 (7)	
H7A	0.6639	0.8255	0.7157	0.024*	
H7B	0.5964	0.8066	0.5742	0.024*	
C8	0.6522 (3)	0.9378 (2)	0.6026 (3)	0.0232 (8)	
H8A	0.6505	0.9505	0.5099	0.028*	
H8B	0.5630	0.9567	0.6332	0.028*	

### Atomic displacement parameters (Å^2^)

**Table:**

	*U*^11^	*U*^22^	*U*^33^	*U*^12^	*U*^13^	*U*^23^
Co	0.0151 (2)	0.0141 (2)	0.0135 (2)	0.00047 (19)	0.00119 (17)	−0.00033 (17)
Cl1	0.0197 (4)	0.0212 (4)	0.0161 (4)	0.0040 (3)	0.0027 (3)	−0.0015 (3)
Cl2	0.0282 (5)	0.0152 (4)	0.0190 (4)	0.0025 (3)	0.0045 (3)	0.0005 (3)
Cl3	0.0165 (4)	0.0196 (4)	0.0175 (4)	−0.0021 (3)	−0.0015 (3)	0.0018 (3)
Cl4	0.0242 (5)	0.0225 (4)	0.0130 (4)	0.0032 (3)	0.0010 (3)	0.0004 (3)
O1	0.0180 (13)	0.0232 (13)	0.0358 (14)	0.0090 (10)	0.0089 (11)	0.0097 (10)
N1	0.0140 (15)	0.0217 (15)	0.0179 (14)	0.0042 (12)	−0.0025 (12)	−0.0014 (11)
C1	0.029 (2)	0.0201 (18)	0.0277 (19)	0.0023 (16)	0.0087 (17)	0.0110 (15)
C2	0.020 (2)	0.0171 (18)	0.0218 (17)	−0.0042 (14)	0.0053 (15)	−0.0008 (14)
C3	0.0257 (19)	0.0131 (17)	0.0146 (15)	−0.0001 (14)	0.0036 (14)	0.0018 (13)
C4	0.0151 (18)	0.0252 (19)	0.0277 (18)	0.0011 (15)	0.0066 (15)	0.0039 (15)
O2	0.0200 (13)	0.0208 (12)	0.0252 (12)	−0.0009 (10)	0.0033 (10)	−0.0135 (10)
N2	0.0224 (16)	0.0127 (14)	0.0194 (14)	0.0002 (12)	0.0002 (12)	0.0008 (11)
C5	0.024 (2)	0.0232 (19)	0.031 (2)	−0.0098 (15)	0.0019 (16)	−0.0087 (15)
C6	0.0123 (18)	0.028 (2)	0.036 (2)	−0.0012 (15)	−0.0037 (16)	−0.0058 (15)
C7	0.0169 (18)	0.0225 (18)	0.0197 (17)	−0.0057 (14)	0.0031 (14)	−0.0019 (14)
C8	0.0177 (18)	0.0231 (19)	0.0287 (19)	−0.0009 (15)	0.0021 (15)	−0.0066 (15)

### Geometric parameters (Å, °)

**Table:**

Co—Cl1	2.2583 (8)	C4—H4A	0.9900
Co—Cl2	2.2644 (8)	C4—H4B	0.9900
Co—Cl4	2.2656 (8)	O2—C8	1.420 (4)
Co—Cl3	2.2818 (8)	O2—C5	1.410 (4)
O1—C1	1.428 (4)	N2—C6	1.486 (4)
O1—C4	1.419 (4)	N2—C7	1.492 (4)
N1—C3	1.488 (4)	N2—H2A	0.9200
N1—C2	1.494 (4)	N2—H2B	0.9200
N1—H1A	0.9200	C5—C6	1.503 (4)
N1—H1B	0.9200	C5—H5A	0.9900
C1—C2	1.507 (4)	C5—H5B	0.9900
C1—H1C	0.9900	C6—H6A	0.9900
C1—H1D	0.9900	C6—H6B	0.9900
C2—H2C	0.9900	C7—C8	1.503 (4)
C2—H2D	0.9900	C7—H7A	0.9900
C3—C4	1.498 (4)	C7—H7B	0.9900
C3—H3A	0.9900	C8—H8A	0.9900
C3—H3B	0.9900	C8—H8B	0.9900
			
Cl1—Co—Cl2	113.65 (3)	O1—C4—H4B	109.4
Cl1—Co—Cl4	106.01 (3)	C3—C4—H4B	109.4
Cl2—Co—Cl4	113.73 (3)	H4A—C4—H4B	108.0
Cl1—Co—Cl3	108.68 (3)	C8—O2—C5	109.5 (2)
Cl2—Co—Cl3	107.50 (3)	C6—N2—C7	111.0 (2)
Cl4—Co—Cl3	107.00 (3)	C6—N2—H2A	109.4
C1—O1—C4	109.9 (2)	C7—N2—H2A	109.4
C3—N1—C2	111.2 (2)	C6—N2—H2B	109.4
C3—N1—H1A	109.4	C7—N2—H2B	109.4
C2—N1—H1A	109.4	H2A—N2—H2B	108.0
C3—N1—H1B	109.4	O2—C5—C6	111.5 (3)
C2—N1—H1B	109.4	O2—C5—H5A	109.3
H1A—N1—H1B	108.0	C6—C5—H5A	109.3
O1—C1—C2	111.9 (2)	O2—C5—H5B	109.3
O1—C1—H1C	109.2	C6—C5—H5B	109.3
C2—C1—H1C	109.2	H5A—C5—H5B	108.0
O1—C1—H1D	109.2	N2—C6—C5	108.8 (3)
C2—C1—H1D	109.2	N2—C6—H6A	109.9
H1C—C1—H1D	107.9	C5—C6—H6A	109.9
N1—C2—C1	109.5 (2)	N2—C6—H6B	109.9
N1—C2—H2C	109.8	C5—C6—H6B	109.9
C1—C2—H2C	109.8	H6A—C6—H6B	108.3
N1—C2—H2D	109.8	N2—C7—C8	109.4 (2)
C1—C2—H2D	109.8	N2—C7—H7A	109.8
H2C—C2—H2D	108.2	C8—C7—H7A	109.8
N1—C3—C4	109.3 (2)	N2—C7—H7B	109.8
N1—C3—H3A	109.8	C8—C7—H7B	109.8
C4—C3—H3A	109.8	H7A—C7—H7B	108.2
N1—C3—H3B	109.8	O2—C8—C7	111.6 (3)
C4—C3—H3B	109.8	O2—C8—H8A	109.3
H3A—C3—H3B	108.3	C7—C8—H8A	109.3
O1—C4—C3	111.0 (3)	O2—C8—H8B	109.3
O1—C4—H4A	109.4	C7—C8—H8B	109.3
C3—C4—H4A	109.4	H8A—C8—H8B	108.0

### Hydrogen-bond geometry (Å, °)

**Table:**

*D*—H···*A*	*D*—H	H···*A*	*D*···*A*	*D*—H···*A*
N1—H1A···Cl2^i^	0.92	2.40	3.275 (3)	159.
N1—H1B···Cl1	0.92	2.38	3.184 (2)	146.
N2—H2A···Cl3	0.92	2.45	3.232 (3)	142.
N2—H2B···Cl3^ii^	0.92	2.37	3.264 (3)	163.
C2—H2C···Cl1^ii^	0.99	2.71	3.603 (3)	151.
C3—H3B···Cl4^i^	0.99	2.77	3.556 (3)	136.
C5—H5A···Cl3^iii^	0.99	2.83	3.767 (3)	158.

Symmetry codes: (i) −*x*+1, −*y*+1, −*z*+1; (ii) *x*, −*y*+3/2, *z*−1/2; (iii) −*x*+2, *y*+1/2, −*z*+3/2.
